# Exploring Protein Space: From Hydrolase to Ligase by Substitution

**DOI:** 10.1093/molbev/msaa215

**Published:** 2020-09-01

**Authors:** Nir Hecht, Caroline L Monteil, Guy Perrière, Marina Vishkautzan, Eyal Gur

**Affiliations:** 1 Department of Life Sciences, Ben-Gurion University of the Negev, Beer-Sheva, Israel; 2 Laboratoire de Biométrie et Biologie Evolutive, Université Claude Bernard – Lyon 1, Villeurbanne, France; 3 The National Institute for Biotechnology in the Negev, Ben-Gurion University of the Negev, Beer-Sheva, Israel

**Keywords:** Dop, PafA, Pup, structure–function, molecular evolution

## Abstract

The understanding of how proteins evolve to perform novel functions has long been sought by biologists. In this regard, two homologous bacterial enzymes, PafA and Dop, pose an insightful case study, as both rely on similar mechanistic properties, yet catalyze different reactions. PafA conjugates a small protein tag to target proteins, whereas Dop removes the tag by hydrolysis. Given that both enzymes present a similar fold and high sequence similarity, we sought to identify the differences in the amino acid sequence and folding responsible for each distinct activity. We tackled this question using analysis of sequence–function relationships, and identified a set of uniquely conserved residues in each enzyme. Reciprocal mutagenesis of the hydrolase, Dop, completely abolished the native activity, at the same time yielding a catalytically active ligase. Based on the available Dop and PafA crystal structures, this change of activity required a conformational change of a critical loop at the vicinity of the active site. We identified the conserved positions essential for stabilization of the alternative loop conformation, and tracked alternative mutational pathways that lead to a change in activity. Remarkably, all these pathways were combined in the evolution of PafA and Dop, despite their redundant effect on activity. Overall, we identified the residues and structural elements in PafA and Dop responsible for their activity differences. This analysis delineated, in molecular terms, the changes required for the emergence of a new catalytic function from a preexisting one.

## Introduction

The concept of “*protein space*” was introduced in 1970 by John Maynard Smith (Maynard Smith 1970) in an attempt to settle the apparent contradiction between evolution by natural selection and the complex nature of the gene-encoded protein (Salisbury 1969). Clearly, for enzymes to evolve and new functions to emerge, changes to the amino acid sequence must take place. However, proteins are of inherent restricted evolvability, as proteins are only marginally stable (ΔΔ*G*_unfolding_ ∼5 to 10 kcal/mol) (DePristo et al. 2005), and about one third of random mutations in proteins have severe effects on their function (>90% loss of activity) (Camps et al. 2007). For natural selection to act as a driving force for molecular evolution, the enzyme catalytic activity must be retained at some level, as an inactive enzyme is a dead end for natural selection. Hence, protein space represents the continuous network of viable sequence combinations via a stepwise mutational process. The mutational trajectory in which protein evolution occurs—while retaining catalytic activity and stability—is complex, given the stochastic nature of mutation and the vast sequence space of proteins. Function-altering mutations are often destabilizing, and additional mutations are required to compensate for this effect. Furthermore, the effect of mutation is not simply additive and could be epistatic in nature; namely, the same mutation could be either neutral, beneficial, or deleterious, depending on the context of the protein sequence. Thus, interactions between mutations pose severe restrictions over evolutionary trajectories (Camps et al. 2007; Kaltenbach and Tokuriki 2014).

**Fig. 1.**
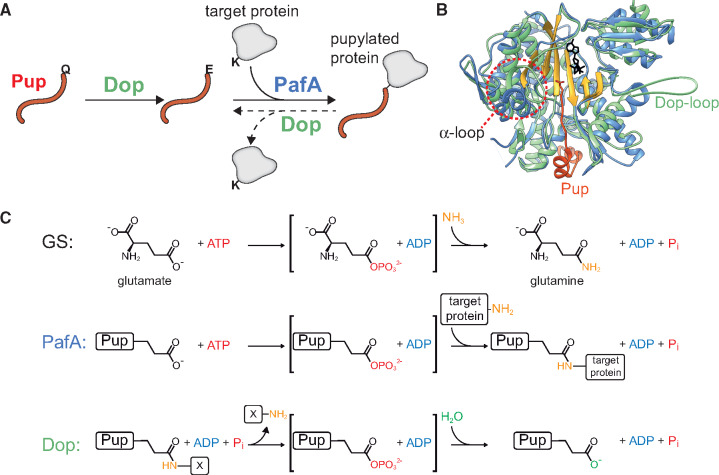
The mycobacterial pupylation pathway. (*A*) Pup is translated with a C-terminal glutamine (Pup^Q^) and its deamidation by Dop converts this glutamine into a glutamate, thus generating Pup^E^. PafA conjugates Pup^E^ to lysine side chains of protein targets, whereas Dop can hydrolyze the isopeptide bond formed by PafA. (*B*) Structural alignment of Dop (green, PDB: 4B0R) (Özcelik et al. 2012) and PafA (blue), in complex with Pup (red) (PDB: 4BJR) (Barandun et al. 2013). Dop and PafA are homologous enzymes that present high structural similarity. Two distinctive differences between the two enzymes are the presence of the Dop-loop in Dop but not in PafA, and the region of the alpha-loop, where an alpha-helix is formed in PafA and a loop in Dop. The illustrated Dop-loop segment was added for visualization purposes only. The active site groove is indicated by gold, and ATP is shown in black. (*C*) Dop and PafA belong to the carboxylate–amine ligase superfamily. Both GS and PafA employ a two-step catalytic mechanism, where ATP is used in the first step to phosphorylate a γ-glutamyl group, followed by ligation of the amine group of a lysine residue (PafA) or ammonia (GS) in the second step. In contrast, Dop hydrolyzes an amide bond using ADP and Pi. X denotes either hydrogen or target protein for deamidation and depupylation, respectively.

Although understanding evolution at the molecular level is a central goal in modern biology, studying evolution involves inherent difficulties, as tracking past events always involves some level of uncertainty. Most research in this field is conducted synthetically, in vitro, using directed evolution, whereas kinetic parameters like *k*_cat_ or *K*_m_ are used as a proxy for organism fitness. Here we describe the evolutionary relationship between two homologous enzymes, Dop and PafA, and demonstrate in molecular detail the changes required for the emergence of a new catalytic function from a preexisting one. Dop and PafA pose an insightful case study, as both rely on similar mechanistic properties, yet catalyze distinct reactions (Striebel et al. 2009; Özcelik et al. 2012). PafA catalyzes the ligation of a small protein tag termed Pup (*P*rokaryotic *u*biquitin-like *p*rotein) to target protein substrates (Guth et al. 2011); Dop removes the tag by hydrolysis of the iso-peptide bond between Pup and the target protein (fig. 1*A*) (Burns et al. 2010). Together, they form the pupylation pathway, a conserved pathway in species belonging to the phyla Actinobacteria and Nitrospira (Iyer et al. 2008). In *Mycobacterium tuberculosis*, pupylation is coupled to regulated protein degradation by the bacterial proteasome, and is essential for virulence of this pathogen (Darwin 2003). In the nonpathogenic model organism *Mycobacterium smegmatis*, the *P*up-*p*roteasome *s*ystem (PPS) plays an important physiological role under nitrogen starvation conditions (Elharar et al. 2014). Since Dop and PafA are the products of natural evolution, they form an advantageous, *bona fide*, experimental system to explore protein space and test the effect of mutation on protein stability, function, and fitness—both biochemically and in the context of the living cell.

The *M. smegmatis* Dop and PafA share 37% identity and 65% similarity; both belong to the carboxylate–amine ligase superfamily and share the glutamine synthetase (GS) fold (fig. 1*B*) (Iyer et al. 2008; Özcelik et al. 2012). Although PafA and Dop clearly had a common ancestor, they present distinct activities with no detectable promiscuous activities (Striebel et al. 2009). In other words, PafA does not perform deamidation and depupylation, whereas Dop cannot pupylate substrates. Very much like GS, PafA catalyzes a two-step reaction where ATP is used in the first step to phosphorylate a γ-glutamyl group, thereby facilitating conjugation to an amine group in the second step alongside the release of a free phosphate. Specifically, PafA phosphorylates Pup C-terminal glutamate in the first step, and proceeds to the conjugation of this activated Pup form with the ε-amino group of a target protein lysine (fig. 1*C*) (Guth et al. 2011). In mycobacteria and some other species, Pup is translated with a C-terminal glutamine (Pup^Q^) rather than a glutamate (Pup^E^) (Pearce et al. 2008). In these cases, Dop is responsible for deamidation of Pup^Q^, leading to the formation of Pup^E^ (fig. 1*A*) (Striebel et al. 2009). Only then can PafA conjugate Pup^E^ to target substrates. Via the same mechanism, Dop can also depupylate an already pupylated protein (fig. 1*C*), albeit slower than it catalyzes deamidation (Elharar et al. 2016; Hecht et al. 2018). It is of note that although PafA and Dop share catalytic properties, the Dop catalytic mechanism is not fully understood. Presumably, it hydrolyzes an ATP molecule and uses the phosphate group for multiple cycles, all the time binding the resulting ADP. In each cycle, the phosphate is used to break the isopeptide bond, thus forming the phospho-acyl Pup intermediate. Next, a water molecule, or more likely a hydroxyl ion, is used to hydrolyze the phosphorylated Pup intermediate, liberating a free Pup^E^ (fig. 1*C*) (Bolten et al. 2017).

Although Dop and PafA present a similar fold, their structures differ significantly in two regions. A region of ∼40 amino acids, termed the Dop-loop, is conserved in Dop, but is absent in PafA orthologs (Özcelik et al. 2012). The second noticeable structural difference between PafA and Dop lies in a region which we termed the “alpha-loop,” as this region forms an alpha-helix in PafA, in contrast to a loop in Dop (fig. 1*B*). Although the Dop-loop and the alpha-loop clearly differentiate between Dop and PafA, they are not essential for catalysis, and switching either of them between the enzymes did not lead to a change in activity (Özcelik et al. 2012). It was later found that the alpha-loop is important for PafA interaction with pupylation targets (Regev et al. 2016), whereas the Dop-loop had been found to allosterically inhibit Dop depupylation activity (Hecht et al. 2020).

Here, we sought to identify the critical differences in amino acid sequence and folding responsible for each distinct activity. We tackled this question initially via analysis of sequence–function relationships, and identified a set of uniquely conserved residues in each enzyme. A follow-up reciprocal mutagenesis of Dop completely abolished the native hydrolase activity, and at the same time yielded a catalytically active Pup-ligase. Mutational analysis, combined with the available structural information, indicated that the alpha-loop conformation is a critical factor that controls the protein function. Further analysis revealed conserved residues to be essential for stabilization of the alternative conformation required for a change in activity, rather than affecting the catalytic mechanism directly. Remarkably, a combinatorial mutant library of the identified residues uncovered multiple mutational paths, each enabling the change of function to occur. Overall, this study highlights, in molecular terms, the changes required for the emergence of a new catalytic function from a preexisting one.

## Results

### Evolutionary Relationship between Dop and PafA

**Fig. 2.**
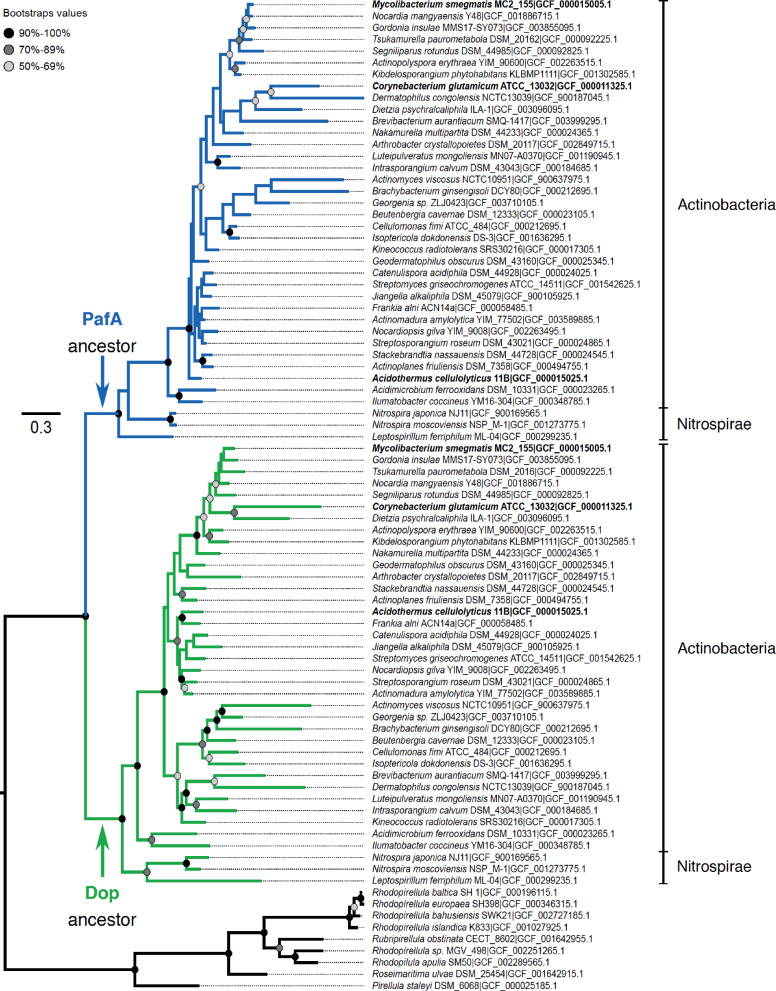
Phylogenetic tree showing the evolutionary relationships between Dop and PafA in the Actinobacteria and Nitrospirae phyla. The tree was built using the Maximum-Likelihood method implemented in IQ-TREE and the model LG+F+R5 for describing amino-acid evolution; 200 replicates of a nonparametric bootstrap approach were conducted to test the robustness of the tree topology and are represented by colored dots. Paralogous sequences related to both Dop (green) and PafA (blue), identified in some Planctomycetes species, were used to root the tree (black). Sequences of the model organisms are in bold; namely *M. smegmatis* strain MC^2^ 155, *A. cellulolyticus* strain 11B and *C. glutamicum* strain ATCC 13032. The branch length represents the number of substitutions per site.

To give some insight into the evolutionary history of Dop and PafA a phylogenetic analysis was performed. Initially, taxa bearing Dop and PafA homologous sequences were identified, via alignment of the *M. smegmatis* strain MC^2^155 Dop and PafA sequences against the refseq_protein database using BLASTP searches. The analysis confirmed that Dop and PafA are largely conserved across the Actinobacteria and Nitrospirae phyla. Homologous sequences of one or both proteins were also detected very sporadically in a few draft genomes within other phyla, like the candidate division NC10, Armatimonadetes, Verrucomicrobia, Nitrospinae, Firmicutes, and Proteobacteria. A single copy of a homolog to both Dop and PafA was identified in some Planctomycetes species and further used as an external group for construction of a maximum likelihood phylogenetic tree. To reliably obtain this, we used the highest quality sequences that also represent the maximum diversity of bacteria having a complete pupylation pathway. We thus selected only complete genomes of the RefSeq database (https://www.ncbi.nlm.nih.gov, last accessed September 1, 2020) available in February 2019. Given the massive number of genomes available, a reduction of the data set was done by selecting randomly only one genome per Actinobacteria family, and per species for the other phyla. The resulting tree built with Dop and PafA sequences indicated that Dop and PafA form two distinct and statistically well-supported clusters that originated from an ancient duplication event (fig. 2). The Planctomycetes paralogous proteins share about 29–31% identity with both Actinobacteria and Nitrospirae PafA and Dop proteins, and their sequence partially aligns with the Dop-loop (MAFFT alignment in supplementary file 1, Supplementary Material online). The data further suggests, given the sporadic co-occurrence of the Pup-ligases and depupylases in phyla other than Actinobacteria, and the current vision of the tree of Bacteria (Hug et al. 2016), that the full pupylation pathway emerged in Actinobacteria and was later horizontally transferred to the ancestor at the origin of the Nitrospirae phylum and to other phyla like Nitrospinae and Proteobacteria.

### Identification of Residues Responsible for an Activity Change

**Fig. 3.**
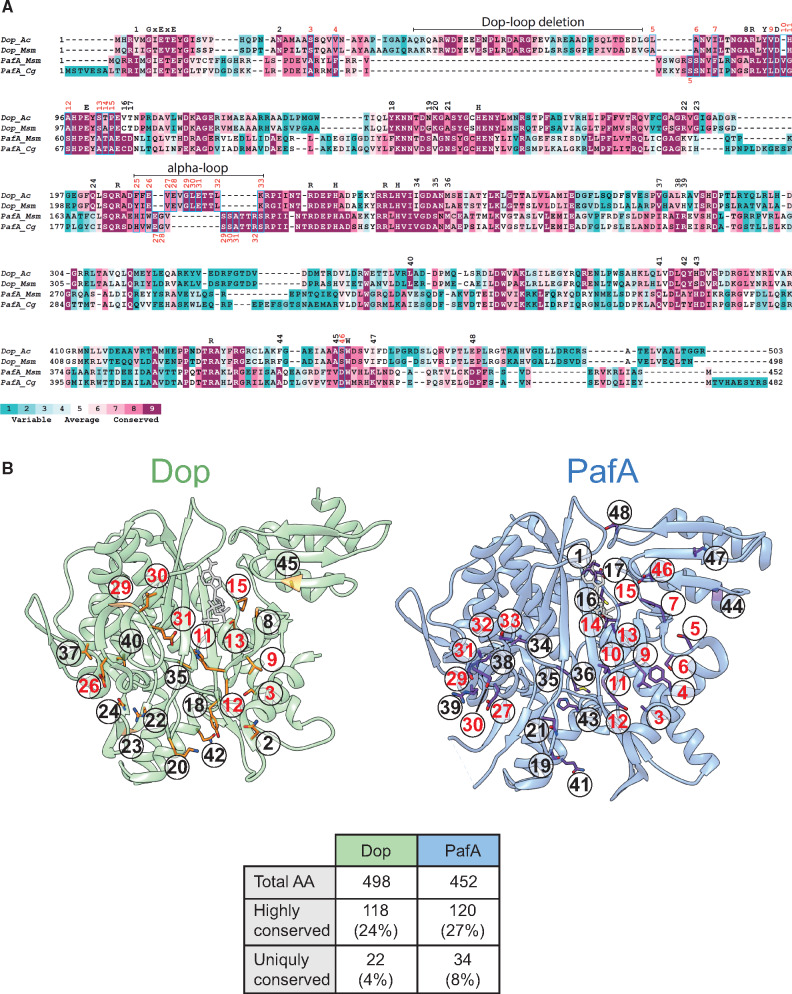
Sequence–structure analysis of Dop and PafA. (*A*) Structure-based sequence alignment of Dop and PafA. The conservation score for each position, calculated separately for either Dop or PafA orthologs by the ConSurf web-server, is color-coded. Uniquely conserved positions in either PafA or Dop are numbered. Positions chosen for subsequent mutagenesis are colored red. Shared conserved residues that take part in binding of the nucleotide or in the course of the reaction are shown above the alignment. Ac, *Acidothermus cellulolyticus*; Msm, *Mycobacterium smegmatis*; Cg, *Corynebacterium glutamicum.* (*B*) The structures of Dop (green, PDB: 4B0R) (Özcelik et al. 2012) and PafA (blue, PDB: 4BJR) (Barandun et al. 2013). Uniquely conserved residues are shown in stick representation and are numbered according to the sequence alignment. The numbers for the residues that were chosen for subsequent mutagenesis are colored red. ATP is colored gray. A quantitative distribution of the highly and uniquely conserved positions is displayed in the table, referring to Dop and PafA of *M. smegmatis*.

To find the residues responsible for the catalytic differences between PafA and Dop, we sought to identify uniquely conserved positions in each enzyme. These were defined as positions conserved in one enzyme but not in the other, or differently conserved in both. We analyzed 2,689 protein sequences belonging to the Pup-ligase/deamidase family, and generated a sequence similarity network (SSN) to categorize each sequence as either a Pup-ligase or a deamidase. The resulting SSN comprised clusters of 377 Dop sequences and 285 PafA sequences (supplementary fig. S1, Supplementary Material online). Multiple sequence alignment (MSA) of the sequences in each cluster was performed, followed by computation of evolutionary conservation score for each position, while taking into account the phylogenetic relations within the alignment (supplementary files 2 and 3, Supplementary Material online). Finally, a structure-based sequence alignment was created via superposition of the Dop and PafA structures (fig. 3*A*). To this end, we relied on the structural information available for the *Acidothermus cellulolyticus* Dop [PDB: 4B0R] (Özcelik et al. 2012) and *Corynebacterium glutamicum* PafA [PDB: 4BJR] (Barandun et al. 2013) structures.

Considering only positions with maximal conservation score, we identified 118 conserved residues in Dop and 120 in PafA (supplementary files 2 and 3, Supplementary Material online). Most of these conserved residues were located at the active site beta-sheet cradle (supplementary fig. S2, Supplementary Material online). These included residues which are conserved not only in Dop and PafA, but rather across the carboxylate–amine ligase superfamily (fig. 3*A*). Examples of such residues are the GhExE (h, hydrophobic; x, any residue) ATP-binding motif and additional residues that were previously shown to be involved in catalysis (Iyer et al. 2008; Özcelik et al. 2012). Importantly, only 22 Dop positions and 34 PafA positions were found to be uniquely conserved, sharing 10 positions that are differently conserved in both enzymes (fig. 3*A* and *B* and supplementary file 4, Supplementary Material online). We regarded these positions as potentially responsible for the catalytic differences between the two enzymes. Noteworthy, seven of the identified positions were located at the active site alpha-loop region, where the structures of Dop and PafA secondary structures differ (fig. 3*A* and *B*).

**Fig. 4.**
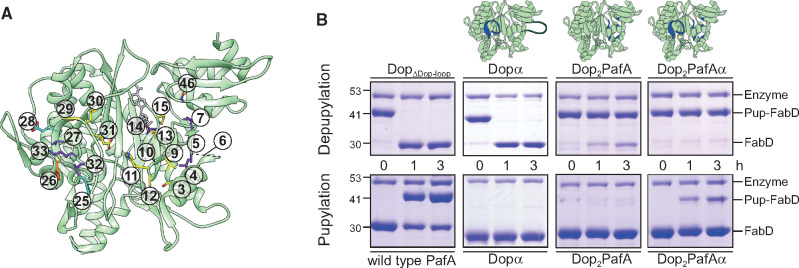
A change of function by 20 mutations. (*A*) The structure of Dop (green, PDB: 4B0R) (Özcelik et al. 2012) with the uniquely conserved positions chosen for mutagenesis, is shown in stick representation and numbered according to the sequence alignment displayed in figure 3*A*. Color: yellow, uniquely conserved position in both enzymes. Purple, residues uniquely conserved in PafA. Orange, uniquely conserved residues in Dop. Cyan, positions chosen for mutagenesis as part of the alpha-loop region. ATP is colored gray. (*B*). Pup-FabD (5 μM) depupylation (Top) and FabD (10 μM) pupylation (Bottom) by the three Dop mutants (1 μM each) with Dop_ΔDop-loop_ and wild type PafA as positive controls. Pup^E^ was used at a concentration of 20 µM. Samples were removed at the indicated time points for SDS–PAGE analysis followed by CBB staining. A cartoon representation of the Dop mutants is depicted for clarification; the location of the mutations is highlighted in blue.

Next, reciprocal mutagenesis was performed on the *M. smegmatis* PafA and Dop. As PafA mutagenesis destabilized the enzyme, we describe here the mutational analysis performed on Dop. To simplify the analysis, uniquely conserved residues that were not located in close proximity to the active site cradle (>20 Å) were filtered, leaving 20 positions in Dop that were selected for reciprocal mutagenesis (fig. 4*A* and table 1). These included nine out of the ten shared positions of both enzymes, eight PafA uniquely conserved positions including one insertion, and one Dop uniquely conserved position. In addition, two positions in the alpha-loop region that were not highly conserved in PafA and Dop were nevertheless chosen for mutagenesis to maintain secondary structure integrity.

**Table 1.** Dop Positions That Were Selected for Reciprocal Mutagenesis.

	Dop			PafA		
No.	*M. smegmatis*	*A. cellulolyticus*	Consurf score	*M. smegmatis*	*C. glutamicum*	Consurf score
3	Ser27	Ser27	**9**	Ala30	Ala37	**9**
4	Val31	Val31	8	Phe34	Phe41	**9**
Δ^a^	Ala43-Val79					
5^a^	Ala81	Leu80	7	Ser43	Ser50	**9**
6^a^	Ala82	Ala81	8	Ser44	Ser51	**9**
7	Ile85	Ile84	8	Phe47	Phe54	**9**
9^b^	Val94	Val93	**9**	Leu56	Leu63	**9**
10^b^	Insertion			Val58	Val65	**9**
11^b^	His96	His95	**9**	Gly59	Gly66	**9**
12^b^	Ala97	Ala96	**9**	Ser60	Ser67	**9**
13	Ser102	Ser101	**9**	Ala65	Ala72	**9**
14	Ala103	Thr102	7	Thr66	Thr73	**9**
15	Pro104	Pro103	**9**	Ala67	Ala74	**9**
25^c^	Tyr209	Phe208	8	His174	His188	8
26^c^	Glu211	Glu210	**9**	Trp176	Trp190	7
27^c^	Val212	Val211	8	Glu177	Glu191	**9**
28^c^	Glu213	Glu212	8	Gly178	Gly192	7
29^c^	Gly215	Gly214	**9**	Ser180	Ser194	**9**
30^c^	Leu216	Leu215	**9**	Ser181	Ser195	**9**
31^c^	Glu217	Glu216	**9**	Ala182	Ala196	**9**
32^c^	Leu220	Leu219	8	Arg185	Arg199	**9**
33^c^	Lys221	Lys220	8	Ser186	Ser200	**9**
46	Ser450	Ser452	8	Asp418	Asp439	**9**

a Ala81,82Ser mutagenesis was performed as part of the Dop-loop deletion (37 a.a.).

b Residues neighboring the catalytic Asp.

c Alpha-loop residues.

Three mutants were designed. The first mutant, Dopα, included only a substitution of the alpha-loop region, comprising nine amino acid substitutions (fig. 4*A* and *B*). The second mutant, Dop_2_PafA, included mutations of 11 positions outside the alpha-loop region; and the third mutant, Dop_2_PafAα, contained all 20 reciprocal mutations. These mutants were initially designed without the Dop-loop, as this region is not essential for Dop catalytic activity (Özcelik et al. 2012; Hecht et al. 2020). Accordingly, a 37 amino acid deletion, which completely removed the loop, was performed while generating the mutants. Eventually, however, the Dopα mutant did contain the Dop-loop, as deletion of this loop destabilized the mutant, rendering it insoluble. The three mutant proteins were expressed in *Escherichia coli* and purified to homogeneity for in vitro depupylation and pupylation assays. For these assays FabD, a *bona fide* substrate, and its pupylated form, Pup-FabD, were used. As FabD and Pup-FabD migrate differently in SDS–PAGE, gel-based assays readily detected pupylation and depupylation in our experimental system. A wild type PafA and a Dop mutant lacking the Dop-loop (Dop_ΔDop-loop_) were used as controls. We found that the Dopα mutant depupylated Pup-FabD as well as Dop_ΔDop-loop_, and did not exhibit any pupylation activity (fig. 4*B*). This result indicated that substitution of only the alpha-region is insufficient for an activity change. The Dop_2_PafA mutant was able to depupylate Pup-FabD, although poorly as compared with the Dop_ΔDop-loop_, and was not able to pupylate FabD. Clearly, the eleven point mutations did not convert Dop into a Pup-ligase. However, when these eleven mutations were combined with the alpha-loop mutations to yield Dop_2_PafAα, the mutant lost its native depupylation activity and functioned as a catalytically active Pup-ligase (fig. 4*B*). Remarkably, 20 mutations were sufficient to completely abolish Dop native activity and to change its catalytic activity from a hydrolase to a ligase.

### The Dop-Loop Contributes to the Change of Function

**Fig. 5.**
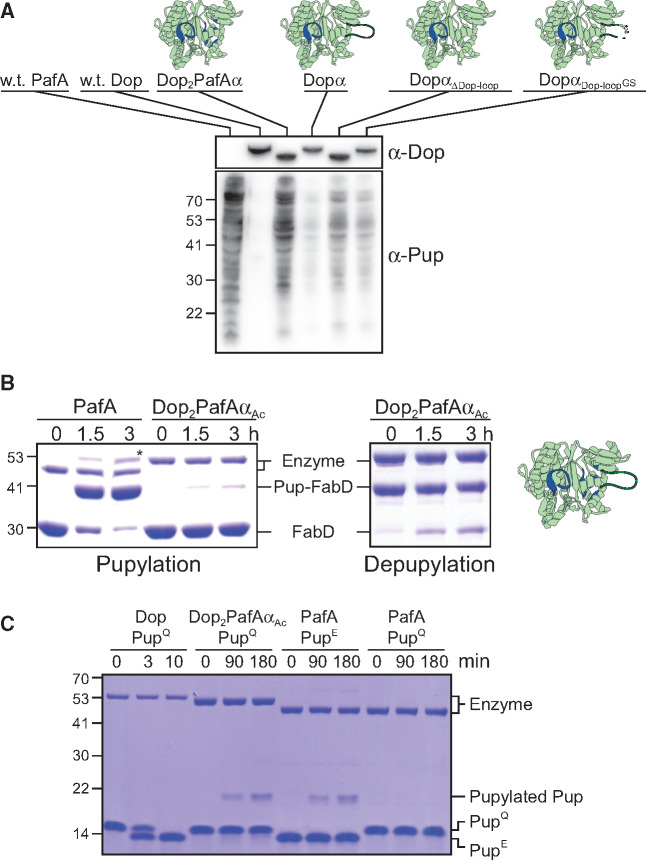
The Dop-loop supports a depupylase activity. (*A*) *Escherichia coli* in vivo pupylation assay of Dopα, Dopα_ΔDop-loop_ and Dopα_Dop-loop_^GS^. Wild type PafA, Dop, and Dop_2_PafAα were used as controls. Western blots using antibodies against Dop and Pup were performed on samples removed from *E. coli* cells expressing the indicated enzymes. Loading controls are presented in supplementary figure S3, Supplementary Material online. (*B*) FabD (10 μM) pupylation (left) and Pup–FabD (5 μM) depupylation (right) by Dop_2_PafAα_AC_ mutant (1 μM) which includes the Dop-loop. Wild type PafA (1 μM) was used as a positive control for pupylation. The asterisk marks the formation of a pupylated PafA band, as PafA is in itself a pupylation substrate (Elharar et al. 2014). A cartoon representation of the Dop mutants is depicted for clarification; the location of the mutations is highlighted in blue, or marked by GS. (*C*). Pup^Q^ (40 μM) deamidation by Dop_2_PafAα_AC_ (1 μM) was examined, with controls comprising reactions using wild type Dop (0.5 μM) and Pup^Q^, wild type PafA (1 μM) and Pup^Q^, and wild type PafA and Pup^E^ (40 μM). Both in *B* and *C*, samples were removed at the indicated time points for SDS–PAGE analysis, followed by Coomassie brilliant blue (CBB) staining.

The mutational analysis described in figure 4*B* did not account for the possibility that, although the Dop-loop is not essential for Dop activities, its deletion nevertheless contributed to the change of function. This flaw resulted from our inability to purify a Dopα mutant lacking the Dop-loop (Dopα_ΔDop-loop_) owing to protein solubility problems. To circumvent this problem, we sought to perform pupylation assays in *E. coli* cells following mild expression of this Dop mutant. Although *E. coli* does not have a PPS, expression of Pup^E^ and PafA in *E. coli* leads to comprehensive pupylation of cellular proteins (Cerda‐Maira et al. 2011). In parallel to the generation of a Dopα_ΔDop-loop_ mutant, we generated and expressed a mutant which we termed Dopα_Dop-loop_^GS^. This mutant had glycine and serine substitutions of conserved residues located at the Dop-loop (figs. 3*A* and 5*A*). As controls, PafA, Dop, Dop2PafAα, and Dopα were expressed. The expression levels of each Dop variant were monitored using Dop-specific antibodies, showing that all variants were well expressed (fig. 5*A*). As Pup^E^ was co-expressed with each tested enzyme, the pupylome (i.e., the pool of pupylated proteins in the cell) levels could be monitored via western blots using antibodies against Pup. As expected, a pupylome was detected upon PafA expression, but not upon expression of wild type Dop. The Dop_2_PafAα mutant produced a pupylome level comparable with that of wild type PafA, whereas the Dopα mutant produced very low pupylation levels. This is consistent with the lack of pupylation observed for the Dopα mutant in vitro (fig. 4*B*). Importantly, Dopα_Dop-loop_^GS^ generated a higher level of pupylome, whereas deletion of the whole Dop-loop (Dopα_ΔDop-loop_) resulted in an even higher pupylome level. Clearly, the Dopα mutant lacking the Dop-loop, with no addition of supporting mutations, was able to perform pupylation in vivo. In other words, the replacement of the alpha-loop region in Dop, combined with the Dop-loop deletion, was sufficient for a change in function to occur. However, this mutant presented lower pupylome levels in comparison with the Dop_2_PafAα mutant, the original mutant that includes 11 supporting mutations in addition to the alpha-loop replacement and the Dop-loop deletion. Therefore, the supporting mutations, although not essential for a change in activity, contributed to the conversion of a depupylase to a Pup-ligase.

Realizing that the Dop-loop presence can inhibit a change in activity, we sought to compare the in vitro activity of Dop_2_PafAα with a similar mutant that also presents the Dop-loop. To avoid solubility problems, we attempted mutagenesis of the Dop ortholog from *A. cellulolyticus* (Dop_Ac_), the ortholog for which a crystal structure is available. Previously, mutational analysis indicated that transplantation of the PafA alpha-loop into Dop_Ac_ did not lead to an activity change (Özcelik et al. 2012). Here, a Dop_2_PafAα_Ac_ mutant was generated, presenting an intact Dop-loop and all the additional 11 supporting mutations (fig. 5*B* and table 1). The Dop_2_PafAα_Ac_ mutant was purified, and its pupylation and depupylation activities were tested in vitro. We found that this mutant could pupylate FabD, albeit very slowly, emphasizing the contribution of the additional supporting mutations for a change in function (fig. 5*B*). Interestingly, the Dop_2_PafAα_Ac_ mutant also retained some depupylation activity, as it was able to depupylate Pup-FabD.

To further understand the Dop-loop contribution to the functional differences between PafA and Dop, a Pup^Q^ deamidation reaction was performed. The product of the deamidation reaction is ${\text{Pup}}_{,}^{E}$ and the two Pup variants migrate slightly differently in SDS–PAGE, thus allowing detection of Pup^Q^ deamidation. Although wild type Dop catalyzed Pup^Q^ deamidation within a few minutes, no Pup^E^ accumulation was observed using the Dop_2_PafAα_Ac_ mutant even after 3 h (fig. 5*C*). At the same time, Dop_2_PafAα_Ac_, in contrast to PafA, was able to use ATP and Pup^Q^ to pupylate Pup. Indeed, using ATP, PafA requires Pup^E^ for pupylation. This result suggests that the Dop_2_PafAα_Ac_ mutant catalyzed a mixed Dop–PafA reaction. Based on the established PafA and Dop mechanisms of action (Guth et al. 2011; Bolten et al. 2017; Hecht et al. 2018), we hypothesize that in the first reaction step, it catalyzed the formation of an acyl-Pup intermediate using Pup^Q^ and ATP, as does wild type Dop. The second step of the reaction proceeded as catalyzed by wild type PafA, with a nucleophilic attack of a ε-amino group of a lysine residue on Pup—the abundant protein target in the reactions depicted in figure 5*C*. As the Dop-loop is present in Dop_2_PafAα_Ac_, we conclude that although the Dop-loop is not required for Dop catalysis, its deletion can contribute to a change of activity by affecting the first step of the reaction.

### The Alpha-Loop Is a Discriminatory Factor

**Fig. 6.**
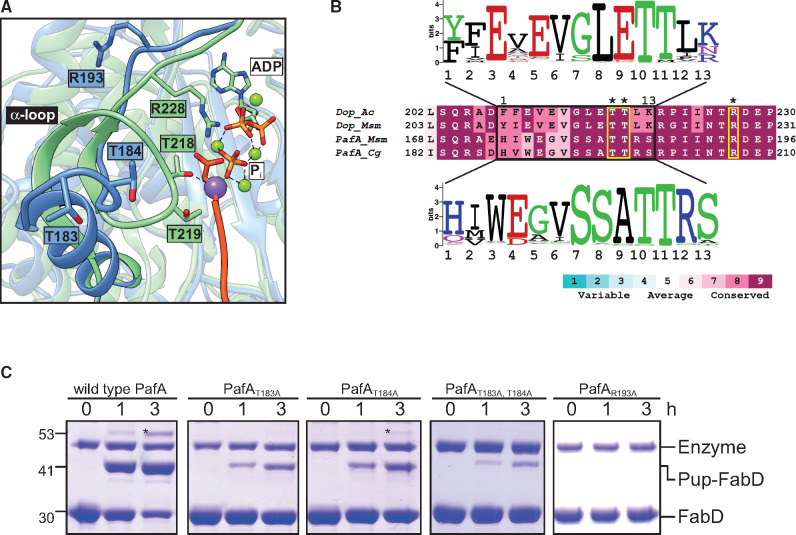
Conserved and functionally important alpha-loop residues are positioned differently in PafA and Dop. (*A*) Structural alignment of the alpha-loop region in Dop (green, PDB: 5LRT) (Bolten et al. 2017) and PafA (blue, PDB: 4B0T) (Özcelik et al. 2012) in complex with Pup (red, PDB: 4BJR) (Barandun et al. 2013) is shown, highlighting the alpha-loop region. The threonine and arginine residues are shown in stick representation as is the Pup C-terminal glutamate. Green spheres represent magnesium ions, and the purple sphere represents a sodium ion. (*B*) Sequence alignment of the alpha-loop region of Dop and PafA, colored according to the conservation score. The residues referred to in the text are surrounded by a yellow square and marked by an asterisk. In addition, a sequence logo of the alpha-loop is presented for each enzyme (Crooks 2004). Polar, green; neutral, purple; basic, blue; acidic, red; hydrophobic, black. (*C*) FabD (10 μM) pupylation by wild type PafA, PafA_T183A_, PafA_T184A_, PafA_T183A_, _T184A_, and PafA_R193A_ (1 µM each) and Pup^E^ (20 µM). Samples were removed at the indicated time points for SDS–PAGE analysis, followed by CBB staining.

Our results thus far indicate that replacement of the alpha-loop region was critical for an activity change (figs. 4*B* and 5*A*). This region in Dop adopts a loop conformation, whereas in PafA an α-helix is formed according to the available crystal structures (fig. 6*A*). We therefore considered the possibility that the alpha-loop conformation determines whether the enzyme functions as a depupylase or as a Pup-ligase. Interestingly, despite the different conformations of the alpha-loop in PafA and Dop, this region presents conserved residues that are identical in both enzymes. Specifically, two threonines and an arginine are highly conserved in both enzymes, and are perfectly aligned in the sequence of Dop and PafA, yet these residues are spatially arranged differently in both enzymes, owing to the different conformation of the alpha-loop region (fig. 6*A* and *B*). In Dop, these residues clearly face the active site, and are potentially involved in catalysis. In PafA, these residues point away from the active site. To test their role in PafA, the two threonines and arginine were mutated to alanines for activity measurements in vitro. The single threonine to alanine mutants (PafA_T183A_, PafA_T184A_) were found active, yet catalyzed FabD pupylation considerably slower than wild type PafA (fig. 6*C*). The double mutant, PafA_T183A_, _T184A_, was found even less active, and no activity could be detected for the arginine to alanine mutant, PafA_R193A_. These results indicate that those alpha-loop residues that are conserved and identical in PafA and Dop are also functionally important, despite their different geometric arrangement in both enzymes. As our data indicate that the alpha-loop is a discriminatory factor that must be altered for an activity change to be achieved, it follows that the alpha-loop conformation, rather than the identity of its functional residues, is a prime factor that differentiates between PafA and Dop.

### Multiple Distinct Mutational Paths Support a Change of Function

Replacement of the alpha-loop resulted in an activity change when combined with supporting mutations that were deduced based on position conservation analysis in PafA and Dop (figs. 3*A* and 4*B*). To determine which of the supporting mutations are indeed essential and responsible for the change in activity, a series of Dop_2_PafAα mutants was created, each presenting a single reversion back to the native state. As some of the mutants proved to be unstable to an extent where it was impossible to express and purify them for in vitro activity assays, in vivo analysis in *M. smegmatis* was carried out. Each Dop mutant was expressed from a plasmid in a *pafA* deletion strain, and the pupylome levels were monitored via western blots using antibodies against Pup. As PafA is the sole Pup-ligase, pupylome accumulation in these strains attested for a Pup-ligase activity of the expressed Dop mutants. To assess the expression levels of the Dop mutants, we relied on a poly-histidine tag present at the N-terminus of each Dop mutant, and performed western blots using antibodies specific for this tag. An empty vector, and vectors expressing wild type Dop and PafA, were used as controls.

**Fig. 7.**
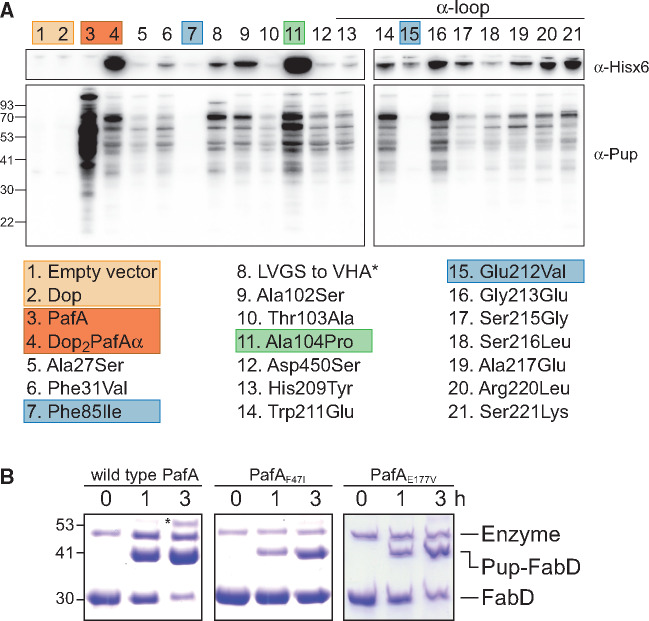
Functionality analysis of Dop_2_PafAα single-reversion mutants. (*A*) A series of polyhistidine-tagged Dop_2_PafAα mutants were expressed in a *M. smegmatis* strain lacking PafA. Each mutant contained a single reversion of a mutation found in Dop_2_PafAα back to the native state. Western blots were performed using antibodies against the polyhistidine tag (upper panel), and against Pup (lower panel). * L94V, V (deletion), G96H, S97A mutant. Loading controls are presented in supplementary figure S3, Supplementary Material online. (*B*) FabD (10 μM) pupylation by wild type PafA, PafA_F47I_, and PafA_E177V_ (1 μM each) and Pup^E^ (20 µM). Samples were removed at the indicated time points for SDS–PAGE analysis followed by CBB staining.

As expected, no pupylation was observed in the negative controls (empty vector, Dop), whereas a high level of pupylation was evident in the clone expressing wild type PafA (fig. 7*A*). Dop_2_PafAα was well expressed in *M. smegmatis*, and gave rise to a clear pupylome, albeit at levels lower than those observed upon PafA expression. In contrast, most of the single-reversion mutants were poorly expressed, suggesting that these reversions destabilized the Dop_2_PafAα mutant. This is consistent with the idea that most of the mutations originally included in the Dop_2_PafAα were stabilizing mutations that were not necessarily required for catalysis per se. Only one reversion mutant, Ala104Pro, exhibited both expression and activity levels higher than the parental mutant, Dop_2_PafAα (fig. 7*A*). Two mutants, Phe85Ile and Glu212Val, lost their pupylation activity to an extent where pupylomes were undetectable. However, since these mutants presented low expression levels, it was difficult to determine whether these positions are functionally important for pupylation. Previous studies did not point to the respective positions in PafA, Phe47, and Glu177 as being functionally important. To further explore the functional importance of these positions in PafA catalysis, reciprocal mutagenesis was performed in the wild type context. Specifically, Phe47 in PafA was mutated to isoleucine, and Glu177 was mutated to valine. The two resulting mutants, PafA_F47I_ and PafA_E177V_, were purified and their activity was tested in vitro. A FabD pupylation assay was performed to test PafA_Phe47Ile_ and PafA_Glu177Val_ activity, and was compared with an assay using wild type PafA. The pupylation activity of both mutants was significantly lower than that of wild type PafA (fig. 7*B*). These results suggest that these positions are functionally important in PafA, and are consistent with their conservation in PafA orthologs (fig. 3*A*).

**Fig. 8.**
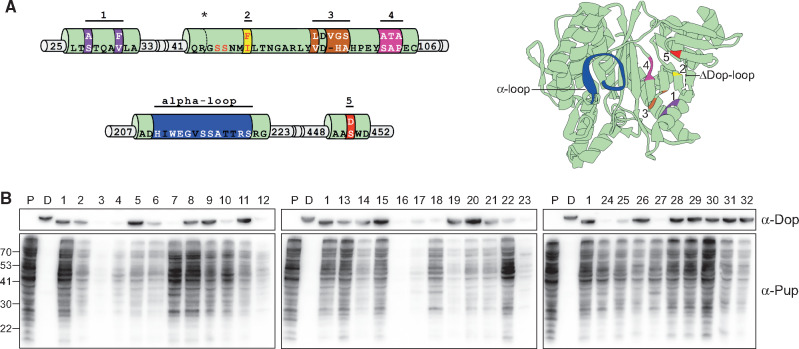
Functionality analysis of a combinatorial Dop mutant library. (*A*) Shown on the left side is a schematic representation of the Dopα_ΔDop-loop_ backbone, with the eleven positions across the five segments containing either the wild type Dop sequence or PafA. The Dop-loop deletion is marked by an asterisk and a dotted line. On the right side, a cartoon representation of the Dop structure is presented, where the segments are color-coded and the alpha-loop region is highlighted in blue. (*B*) Each mutant and Pup^E^ was expressed in *E. coli*, and western blot analysis was performed using antibodies against Dop (upper panel) and against Pup (lower panel). P: PafA, D: Dop, no. 1: Dop_2_PafAα. Loading controls are presented in supplementary figure S3, Supplementary Material online.

To determine the minimal set of supporting mutations that can support a change in activity, we created a combinatorial mutant library using a Dop that presents the alpha-loop as a backbone for addition of mutations. This backbone also lacked the Dop-loop, as in the previous mutational analysis performed in *M. smegmatis* (fig. 7*A*). As 11 positions were mutated alongside the alpha-loop region in Dop_2_PafAα, and as each position can accommodate either a PafA or Dop residue, there are 2^11^ = 2,048 possible combinations of supporting mutations. To simplify the analysis, the supporting mutations were divided into five different segments, with each segment presenting either the Dop or PafA sequence (fig. 8*A*). Accordingly, a total of 2^5^ = 32 mutants were generated, and their activity was tested in vivo. This time, the assays were performed in *E. coli* rather than in *M. smegmatis* to allow for a more rapid and convenient analysis. Pup^E^ was expressed with each of the 32 Dop mutants, whereas PafA, Dop, and Dop_2_PafAα were expressed as controls (fig. 8*B* and table 2). Western blots using antibodies against Pup and Dop were performed to assess the levels of the pupylomes and of the expressed Dop mutants, respectively.

**Table 2.** List of the Dop Combinatorial Mutant Library.

No.	1	2	3^a^	4	5
1^b^	S27A, V31F	I85F	VHA to LVGS	S102A, A103T, P104A	S450D
2				S102A, A103T, P104A	
3			VHA to LVGS	S102A, A103T, P104A	
4	S27A, V31F			S102A, A103T, P104A	
5		I85F		S102A, A103T, P104A	
6				S102A, A103T, P104A	S450D
7	S27A, V31F		VHA to LVGS		
8		I85F	VHA to LVGS		
9	S27A, V31F	I85F			
10	S27A, V31F				S450D
11		I85F			S450D
12	S27A, V31F		VHA to LVGS	S102A, A103T, P104A	
13		I85F	VHA to LVGS	S102A, A103T, P104A	
14	S27A, V31F	I85F		S102A, A103T, P104A	
15	S27A, V31F	I85F	VHA to LVGS		
16			VHA to LVGS	S102A, A103T, P104A	S450D
17	S27A, V31F			S102A, A103T, P104A	S450D
18	S27A, V31F		VHA to LVGS		S450D
19		I85F		S102A, A103T, P104A	S450D
20	S27A, V31F	I85F			S450D
21	S27A, V31F	I85F		S102A, A103T, P104A	S450D
22		I85F	VHA to LVGS	S102A, A103T, P104A	S450D
23	S27A, V31F		VHA to LVGS	S102A, A103T, P104A	S450D
24			VHA to LVGS		
25	S27A, V31F				
26					S450D
27			VHA to LVGS		S450D
28		I85F	VHA to LVGS		S450D
29	S27A, V31F	I85F	VHA to LVGS		S450D
30	S27A, V31F	I85F	VHA to LVGS	S102A, A103T, P104A	
31^b^					
32		I85F			

a V94L, V (insertion), H96G, A97S.

b Dop_2_PafAα (no. 1), Dopα_ΔDop-loop_ backbone (no. 31).

Noticeably, no strong correlation was observed between the mutant Dop expression level and its Pup-ligase activity. This was evident also from the in vivo assay presented in figure 7*A*. Clearly, an enzyme stability and its activity are not tightly linked in the protein space. From the 32 mutants tested, some combinations of mutations resulted in an activity level substantially lower than that observed for the Dopα_ΔDop-loop_ backbone (no. 31). For instance, mutants number 17 and 19 presented very weak pupylation activity (fig. 8*B* and table 2). At the other extreme, four mutants generated pupylome levels comparable with those observed for the Dop_2_PafAα, and included the smallest number of supporting mutations (fig. 8*B* and table 2). These four mutants are no. 7 (S27A, V31F, VHA to LVGS), no. 8 (VHA to LVGS, I85F), no. 9 (S27A, V31F, I85F), and no. 10 (S27A, V31P, S450D). Each included mutations across two segments, suggesting that mutation of only one segment could not effectively support a change in activity. Importantly, the results indicate that alternative mutational paths can support a change in function. Indeed, the four mutants did not share a specific mutation in common, but rather presented different combinations, with each effectively supporting a change in function. This analysis demonstrates that multiple mutational paths were combined in the evolution of PafA and Dop, despite their redundant effect on activity.

## Discussion

**Fig. 9.**
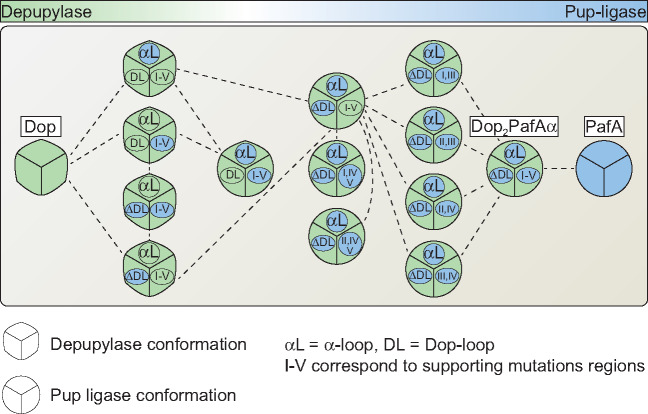
A transition of activity in a continuous protein space. Conversion of a deamidase/depupylase to a Pup-ligase requires a change in conformation. This conformational change can be achieved by substitution of the alpha-loop region, in combination with substitution of either the Dop-loop or different combinations of supporting mutations. These mutations were clustered into regions denoted I–V. The color ruler at the top panel represents activity gradient of the depupylase (green) and of the ligase (blue). The mixed-conformations variant can perform both activities, as did Dop_2_PafAα_Ac_ in this study. The dashed lines denote possible mutational pathways that allow conversion from a depupylase to a ligase and vice versa.

Dop and PafA are close homologs that catalyze opposite reactions. One is a hydrolase; the other a ligase (Striebel et al. 2009; Özcelik et al. 2012). Here, we were able to identify the conserved residues in Dop and PafA that are responsible for the functional differences between these enzymes. Generating Dop_2_PafAα, we converted Dop into a Pup-ligase, whereas the intermediate mutants between Dop and Dop_2_PafAα maintained their depupylation activity (fig. 9). This suggests that along the mutational pathway of an enzyme, a catalytic change can occur following a mutational threshold, namely after a critical number of mutations have accumulated, rather than gradually. Our attempts to convert PafA to a hydrolase via reciprocal mutagenesis were not successful. This implies that the changes that were sufficient for a change in Dop activity are not simply reciprocal, and additional or different changes must be made to transform PafA into a hydrolase.

Dop and PafA evolved from duplication of a gene encoding an ancestral enzyme. According to the current view of protein evolution, it is most likely that the ancestral protein have been promiscuous, and the specific pupylation and depupylation activities evolved by sub-functionalization (Conant and Wolfe 2008). Since PafA catalyzes an activity that is essential for the pupylation pathway function, it is more likely that the ancestor had a Pup-ligase activity and presented a promiscuous Dop-like activity. This view of Dop and PafA evolution is also consistent with their belonging to the GS fold, or more specifically to the carboxylate–amine ligase superfamily. Other members of the superfamily include classical GS and two families of γ-glutamyl-cysteine synthetases (GCS1 and GCS2) (Iyer et al. 2008, 2009). However, the Dop catalytic mechanism diverged from enzymes in the superfamily in two major aspects. Although Dop does bind and uses ATP for the first step of the reaction to generate an acyl-phosphate intermediate, it uses the resulting ADP and Pi for multiple catalytic cycles (fig. 1*C*) (Bolten et al. 2017). Although this process is still unclear, our results suggest the involvement of the conserved residues located at the Dop-loop in the unusual catalytic mechanism utilized by Dop. Secondly, the use of a water molecule instead of an amine group as a nucleophile, in the second part of the reaction, is unique and not known in other members of the superfamily. When considering the known enzymatic mechanisms for hydrolysis of an amide bond (as in proteolysis), Dop stands out as an unusual amidase. At first glance, such an unusual solution for catalysis of a widespread hydrolytic process may seem odd. However, when considering the evolutionary lineage of Dop, modifying an existing scaffold that already binds Pup stands to reason.

It appears that most of the mutations required for the change in Dop function were necessary for the mutant protein stability, rather than catalysis. Accordingly, single position reversions performed on Dop_2_PafAα resulted in most cases in reduced expression levels, which we attribute to reduced stability. From the structural and biochemical point of view, our results demonstrate that although the region of the alpha-loop contains catalytic residues that are highly conserved in both enzymes, a conformational change must take place to convey an activity change. Although structural information on the alpha-loop in the Dop_2_PafAα mutant is currently unavailable, deduction from the available Dop and PafA crystal structures in combination with our biochemical and mutational analysis led us to propose that the mutations in Dop_2_PafAα indeed resulted in a structural change of the alpha-loop conformation. Changing the region of the alpha-loop alone is not sufficient for that change to take place, and it must be accompanied by additional point mutations, supposedly to stabilize the needed conformation, demonstrating an epistatic effect between the alpha-loop residues and the supporting mutations. When the supporting mutations were added combinatorically, we found that a minimum of three out of the eleven mutations are required to support a change of function, and that different distinct mutational paths enabled the change, demonstrating a higher than expected probability of change. All of the supporting mutations positions were highly conserved in PafA, however based on our results not all of them are needed to support a Pup-ligase activity. At most, one would expect some of these positions to show a co-evolution relationship rather than been fully conserved. Hence, it seems that multiple mutational paths were combined in PafA evolution. This could be considered beneficial in terms of evolvability, however it is not clear what could be the selective pressure for this kind of redundancy and how general is this phenomena in protein evolution.

This study demonstrates the changes required in protein space for a new catalytic activity to evolve from a preexisting one. We identified a secondary conserved network of positions that are responsible for the change in activity, and by doing so explored the evolutionary consequences of the complex interplay that takes place between catalytic residues and the “static” protein scaffold that accommodates them. We conclude this discussion with a few sentences from the original paper that introduced the concept of protein space: “Some questions about molecular evolution can be formulated more clearly in terms of a protein space. For example: (i) Are all existing proteins part of the same continuous network, and if so, have they all been reached from a single starting point? (ii) How often, if ever, has evolution passed through a nonfunctional sequence?” (Maynard Smith 1970).

## Materials and Methods

### Bacterial Strains and Growth Conditions

*Mycobacterium smegmatis* MC^2^155 (wild type and mutants) cultures were grown in Middlebrook 7H9 broth containing 0.05% (v/v) Tween-80 and 0.4% (v/v) glycerol at 30 °C. Solid media was prepared using Middlebrook 7H10 supplemented with 0.4% glycerol. *Escherichia coli* ER2566 (New England Biolabs) was used for all cloning procedures and was grown using typical procedures in LB broth and plates at 37 °C. For the *M. smegmatis* in vivo pupylation assay, plasmid pMV206 (Stover et al. 1991) was used for cloning and expression of wild type PafA, Dop, and Dop mutants in a *M. smegmatis* Δ*paf* strain, under the transcriptional control of the *hsp60* promoter. Cultures harboring pMV206 were grown with kanamycin (10 µg/ml). For pupylation assays in *E. coli*, plasmid pBAD24 (Guzman et al. 1995) was used to express Pup^E^ under the control of the arabinose operon, and plasmid pCL1920 (Lerner and Inouye 1990) was used to express wild type PafA, Dop, and Dop mutants under the control of the *lac* promoter–operator. Cultures harboring pBAD24 and pCL1920 were grown with ampicillin (100 µg/ml) and spectinomycin (50 µg/ml), respectively.

### Phylogenetic Analysis

The *hmmsearch* program of the HMMER 3.2.1 software (Eddy 2011; Mistry et al. 2013) and the hidden Markov model (HMM) profiles TIGR03688 and TIGR03686 available in TIGRFAM database (Haft 2003) were initially used to extract Dop and PafA orthologous proteins, respectively. However, we later observed incongruencies in alignments and concluded that the profiles were not discriminative enough to clearly distinguish both paralogs. We thus built HMM profiles in this study with the *hmmbuild* program using Dop and PafA sequences of model organisms. These 20–30 sequences, aligned using the MAFFT v7.313 software (Katoh and Standley 2013), represent several phyla and were unambiguously annotated using the MicroScope annotation platform as Dop or PafA (Vallenet et al. 2009, 2017). Built HMM profiles and alignments are given in supplementary materials. For each genome, only the most significant hit was retained, setting an expectation *E* value threshold of 1e−100. One copy of Dop and PafA was recovered from each genome, aligned using MAFFT and the –lensi option for higher accuracy, and trimmed with the Gblocks software with less stringent parameters (Castresana 2000).

A Maximum-Likelihood tree was built with the IQ-TREE software (Nguyen et al. 2015) and the model LG+F+R5 for describing amino-acid evolution, selected using ModelFinder (Kalyaanamoorthy et al. 2017) and the BIC criterion; 200 replicates of a nonparametric bootstrap approach were conducted to test the robustness of the tree topology. All known proteins in the γ-glutamyl-cysteine synthetases families were too divergent to be used here as an external group. Lowering the expectation E-value threshold to 1e−10, we detected a single copy of a paralogous protein close enough to both Dop and PafA in some Planctomycetes species. This set of single copy PafA-/Dop-related proteins was used as an external group to attest to the duplication event and the ancestry of the indels of Dop and PafA.

### Identification of Uniquely Conserved Positions

Using enzyme function initiative-enzyme similarity tool (EFI-EST) (Gerlt et al. 2011, 2015) web server (http://enzymefunction.org, last accessed September 1, 2020), 2,689 sequences belonging to the Pup-ligase/deamidase family were collected from InterPro (Finn et al. 2017) (IPR004347, IPR022279, IPR022366) and used to generate a SSN (Atkinson et al. 2009; Brown and Babbitt 2014). The resulting SSN was plotted and analyzed using Cytoscape (Shannon et al. 2003; Smoot et al. 2011). The sequences that were clustered together under alignment score of 100 (377 Dop sequences and 285 PafA sequences) were used separately to create a MSA using Jalview (Waterhouse et al. 2009; Troshin et al. 2011) (http://jalview.org, last accessed September 1, 2020) and the Clustal Omega algorithm (Sievers et al. 2011). The resulting MSA was used with ConSurf (Glaser et al. 2003; Landau et al. 2005; Ashkenazy et al. 2010, 2016; Celniker et al. 2013) web server (http://consurf.tau.ac.il/2016, last accessed September 1, 2020) to compute evolutionary conservation for each amino acid based on the phylogenetic relations between sequences. Finally, a sequence alignment from a structural superposition of the solved Dop *A. cellulolyticus* [PDB: 4B0R] (Özcelik et al. 2012) and PafA *C. glutamicum* [PDB: 4BJR] (Barandun et al. 2013) structures was created using UCSF Chimera (Pettersen et al. 2004), Match-Align (Meng et al. 2006).

### Protein Expression and Purification

All proteins used in this study were recombinant *M. smegmatis* proteins, unless stated otherwise. For Pup purification, *pup* was cloned into plasmid pSH21 in fusion with the DNA encoding human titin-I27 and a TEV protease recognition sequence (His_6_-I27-TEV-Pup). Expression was at 30 °C, and Ni^2+^-NTA purification was carried out according to a standard protocol. Following TEV cleavage, a buffer exchange step was carried out, and the His_6_-I27-TEV portion of the chimera was removed by loading the solution onto a Ni^2+^-NTA column. The flow-through was collected, and Pup was further purified on a C18 reverse phase column, lyophilized, and resuspended in 50 mM Hepes, pH 7.5, 50 mM NaCl.

All Dop variants were expressed in *E. coli* strain ER2566 from plasmid pET11a (with a C-terminal polyhistidine tag) or from plasmid pSH21 (N-terminal polyhistidine tag) under the transcriptional control of the T7 promoter. Following induction with IPTG, the cultures were incubated overnight at 18 °C. Cells were lysed by sonication, and purification using Ni^2+^-NTA-agarose (Qiagen) was carried out according to a standard protocol, except that for purification of *M. smegmatis* Dop variants, buffers contained 10% glycerol (v/v). A second size exclusion chromatography purification step relied on a Superdex 200 column (GE Healthcare). For the *M. smegmatis* Dop variants, the buffer used for purification contained 50 mM Hepes, pH 7.5, 150 mM KCl, 20 mM MgCl_2_, 10% (v/v) glycerol and 1 mM DTT. For purification of *A. cellulolyticus* Dop, the buffer contained 50 mM Hepes, pH 8.0, 300 mM NaCl, 20 mM MgCl_2_, and 1 mM DTT.

PafA carried N-terminal polyhistidine tag and was at 30 °C expressed in *E. coli* strain ER2566 from plasmid pSH21 under the transcriptional control of the T7 promoter. Cells were lysed by sonication, and purification using Ni^2+^-NTA-agarose (Qiagen) was carried out according to a standard protocol. Purification Ni^2+^-NTA buffers contained 10% glycerol (v/v). As a consequent purification step, a Superdex 200 size exclusion column (GE Healthcare) equilibrated with 50 mM Hepes, pH 7.5, 500 mM NaCl, and 10% (v/v) was used. The same procedure was used for PanB purification, except that the Superdex 200 size exclusion column (GE Healthcare) was equilibrated with 50 mM Hepes, pH 7.5, 150 mM NaCl, and 10% (v/v). For IdeR purification, the same procedure was used, with a buffer 50 mM Hepes, pH 7.5, 150 mM NaCl for Superdex 200 size exclusion column (GE Healthcare) equilibration. N-terminal polyhistidine tagged *M. tuberculosis* FabD that presents arginine substitutions of lysines 35, 122, and 291 was cloned following the same protocol used for IdeR purification.

For generation and purification of pupylated PanB, IdeR, and FabD, a *C. glutamicum* PafA (cgPafA) was used that presents an N-terminal polyhistidine tag followed by a TEV protease sequence. cgPafA was purified using the same protocol that was used for purification of *M. smegmatis* PafA, except following elution from the Ni^2+^-NTA beads, the imidazole in the buffer was removed via a buffer exchange step using a PD10 column (GE Healthcare), and the TEV protease was added at a TEV/PafA ratio of 1:100 (w/w). Following a 6-h incubation, the protein solution was loaded onto a prewashed Ni^2+^-NTA column, and the cgPafA-containing flow-through was collected and loaded onto a Superdex 200 column (GE Healthcare) prewashed with a buffer containing 50 mM Hepes pH 7.5, 500 mM NaCl, and 1 mM DTT. PanB, IdeR, and FabD were expressed and purified as described above. However, following elution from the Ni^2+^-NTA beads, the buffers were exchanged using PD10 columns (GE Healthcare) into pupylation buffers. For IdeR and FabD, a pupylation buffer lacking glycerol was used. Next, cgPafA and Pup^E^ were added to a final concentration of 2.5 and 200 μM, respectively. Following a 6-h incubation at 30 °C, standard Ni^2+^-NTA purifications were performed to remove cgPafA and Pup^E^, as these proteins lack a polyhistidine tag. The eluted pupylated proteins were further purified by size-exclusion chromatography using a Superdex 200 column (GE Healthcare) prewashed with a buffer containing 25 mM Hepes pH 7.5 and 300 mM NaCl. For PanB, glycerol (10% v/v).

### Multiple Site-Directed Mutagenesis

The QuikChange Lightning Multi Site-Directed Mutagenesis kit (Agilent Technologies) was used to create the 32 Dop combinatorial mutants.

### Activity Assays

The buffer used for all in vitro reactions contained 50 mM Hepes (pH 7.5), 20 mM MgCl_2_, 150 mM KCl, 1 mM DTT, and 10% (v/v) glycerol. Samples were analyzed by electrophoresis on a 12% polyacrylamide Bis-Tris gel followed by Coomassie brilliant blue (CBB) staining. Pupylation, depupylation and deamidation assays were performed in a buffer containing ATP (2 mM) at 30 °C.

For in vivo activity assays, *E. coli* cultures harboring plasmids pBAD24 and pCL1920 were grown overnight (∼20 h) at 30 °C in 5 ml of auto induction media LB broth base (FORMEDIUM) supplemented with 1% glycerol (v/v) and 0.2% arabinose (v/v). *Escherichia coli* and *M. smegmatis* lysates were prepared by sonication of cell pellets in microcentrifuge tubes containing 0.5 ml of 1 mM Tris-HCl, pH 8.0, 1 mM EDTA. Cell debris was removed by centrifugation (18,000 × g, 4 °C) for 10 min. Protein content in each sample was determined using Pierce BCA protein assay kit (Thermo scientific). Equal protein amounts were loaded onto SDS–PAGE for electrophoretic separation, followed by transfer onto PVDF membranes and immuno-detection using standard procedures. As a final step after completion of immunodetection, probed membranes were stained by CBB to verify equal loading and transfer of proteins in each lane.

### Structural Alignment

Molecular graphics and analyses were performed with the UCSF Chimera package (Pettersen et al. 2004).

## Supplementary Material

Supplementary data are available at *Molecular Biology and Evolution* online.

## Supplementary Material

msaa215_Supplementary_Data

## References

[msaa215-B1] Ashkenazy H , Abadi S, Martz E, Chay O, Mayrose I, Pupko T, Ben-Tal N. 2016. ConSurf 2016: an improved methodology to estimate and visualize evolutionary conservation in macromolecules. Nucleic Acids Res. 44(W1):W344–350. 27166375 10.1093/nar/gkw408 PMC4987940

[msaa215-B2] Ashkenazy H , Erez E, Martz E, Pupko T, Ben-Tal N. 2010. ConSurf 2010: calculating evolutionary conservation in sequence and structure of proteins and nucleic acids. Nucleic Acids Res. [Internet] 38(Web Server):W529–533. 10.1093/nar/gkq399 PMC2896094 20478830

[msaa215-B3] Atkinson HJ , Morris JH, Ferrin TE, Babbitt PC. 2009. Using sequence similarity networks for visualization of relationships across diverse protein superfamilies. Jordan IK, editor. PLoS One [Internet] 4(2):e4345. 10.1371/journal.pone.0004345 PMC2631154 19190775

[msaa215-B4] Barandun J , Delley CL, Ban N, Weber-Ban E. 2013. Crystal structure of the complex between prokaryotic ubiquitin-like protein and its ligase PafA. J Am Chem Soc. 135(18):6794–6797. 23601177 10.1021/ja4024012

[msaa215-B5] Bolten M , Vahlensieck C, Lipp C, Leibundgut M, Ban N, Weber-Ban E. 2017. Depupylase Dop requires inorganic phosphate in the active site for catalysis. J Biol Chem. 292(10):4044–4053. 28119453 10.1074/jbc.M116.755645 PMC5354516

[msaa215-B6] Brown SD , Babbitt PC. 2014. New insights about enzyme evolution from large scale studies of sequence and structure relationships. J Biol Chem. 289(44):30221–30228. 25210038 10.1074/jbc.R114.569350 PMC4215206

[msaa215-B7] Burns KE , Cerda-Maira FA, Wang T, Li H, Bishai WR, Darwin KH. 2010. “Depupylation” of prokaryotic ubiquitin-like protein from mycobacterial proteasome substrates. Mol Cell 39(5):821–827. 20705495 10.1016/j.molcel.2010.07.019 PMC2939144

[msaa215-B8] Camps M , Herman A, Loh E, Loeb LA. 2007. Genetic constraints on protein evolution. Crit Rev Biochem Mol Biol. [Internet] 42(5):313–326. 10.1080/10409230701597642 PMC3825456 17917869

[msaa215-B9] Castresana J. 2000. Selection of conserved blocks from multiple alignments for their use in phylogenetic analysis. Mol Biol Evol. [Internet] 17(4):540–552. 10.1093/oxfordjournals.molbev.a026334 10742046

[msaa215-B10] Celniker G , Nimrod G, Ashkenazy H, Glaser F, Martz E, Mayrose I, Pupko T, Ben-Tal N. 2013. ConSurf: using evolutionary data to raise testable hypotheses about protein function. Isr J Chem. 53(3–4):199–206.

[msaa215-B11] Cerda‐Maira FA , McAllister F, Bode NJ, Burns KE, Gygi SP, Darwin KH. 2011. Reconstitution of the *Mycobacterium tuberculosis* pupylation pathway in *Escherichia coli*. EMBO Rep. 12(8):863–870. 21738222 10.1038/embor.2011.109 PMC3147258

[msaa215-B12] Conant GC , Wolfe KH. 2008. Turning a hobby into a job: how duplicated genes find new functions. Nat Rev Genet. 9(12):938–950. 19015656 10.1038/nrg2482

[msaa215-B13] Crooks GE. 2004. WebLogo: a sequence logo generator. Genome Res. [Internet] 14(6):1188–1190. 10.1101/gr.849004 PMC419797 15173120

[msaa215-B14] Darwin KH. 2003. The proteasome of *Mycobacterium tuberculosis* Is required for resistance to nitric oxide. Science (80-.) [Internet] 302(5652):1963–1966. 10.1126/science.1091176 14671303

[msaa215-B15] DePristo MA , Weinreich DM, Hartl DL. 2005. Missense meanderings in sequence space: a biophysical view of protein evolution. Nat Rev Genet. 6(9):678–687. 16074985 10.1038/nrg1672

[msaa215-B16] Eddy SR. 2011. Accelerated profile HMM searches. Pearson WR, editor. PLoS Comput Biol. 7(10):e1002195. 22039361 10.1371/journal.pcbi.1002195 PMC3197634

[msaa215-B17] Elharar Y , Roth Z, Hecht N, Rotkopf R, Khalaila I, Gur E. 2016. Posttranslational regulation of coordinated enzyme activities in the Pup-proteasome system. Proc Natl Acad Sci U S A. 113(12):E1605–1614. 26951665 10.1073/pnas.1525185113 PMC4812726

[msaa215-B18] Elharar Y , Roth Z, Hermelin I, Moon A, Peretz G, Shenkerman Y, Vishkautzan M, Khalaila I, Gur E. 2014. Survival of mycobacteria depends on proteasome-mediated amino acid recycling under nutrient limitation. EMBO J. 33(16):1802–1814. 24986881 10.15252/embj.201387076 PMC4195762

[msaa215-B19] Finn RD , Attwood TK, Babbitt PC, Bateman A, Bork P, Bridge AJ, Chang H-Y, Dosztányi Z, El-Gebali S, Fraser M, et al 2017. InterPro in 2017—beyond protein family and domain annotations. Nucleic Acids Res. 45(D1):D190–199. 27899635 10.1093/nar/gkw1107 PMC5210578

[msaa215-B20] Gerlt JA , Allen KN, Almo SC, Armstrong RN, Babbitt PC, Cronan JE, Dunaway-Mariano D, Imker HJ, Jacobson MP, Minor W, et al 2011. The enzyme function initiative. Biochemistry [Internet] 50(46):9950–9962. 10.1021/bi201312u PMC3238057 21999478

[msaa215-B21] Gerlt JA , Bouvier JT, Davidson DB, Imker HJ, Sadkhin B, Slater DR, Whalen KL. 2015. Enzyme function initiative-enzyme similarity tool (EFI-EST): a web tool for generating protein sequence similarity networks. Biochim Biophys Acta [Internet] 1854(8):1019–1037. 10.1016/j.bbapap.2015.04.015 PMC4457552 25900361

[msaa215-B22] Glaser F , Pupko T, Paz I, Bell RE, Bechor-Shental D, Martz E, Ben-Tal N. 2003. ConSurf: identification of functional regions in proteins by surface-mapping of phylogenetic information. Bioinformatics 19(1):163–164. 12499312 10.1093/bioinformatics/19.1.163

[msaa215-B23] Guth E , Thommen M, Weber-Ban E. 2011. Mycobacterial ubiquitin-like protein ligase PafA follows a two-step reaction pathway with a phosphorylated Pup intermediate. J Biol Chem. 286(6):4412–4419. 21081505 10.1074/jbc.M110.189282 PMC3039397

[msaa215-B24] Guzman LM , Belin D, Carson MJ, Beckwith J. 1995. Tight regulation, modulation, and high-level expression by vectors containing the arabinose PBAD promoter. J Bacteriol. [Internet] 177(14):4121–4130. 10.1128/jb.177.14.4121-4130.1995 PMC177145 7608087

[msaa215-B25] Haft DH. 2003. The TIGRFAMs database of protein families. Nucleic Acids Res. [Internet] 31(1):371–373. 10.1093/nar/gkg128 PMC165575 12520025

[msaa215-B26] Hecht N , Becher M, Korman M, Vishkautzan M, Gur E. Forthcoming 2020. Inter‐ and intramolecular regulation of protein depupylation in *Mycobacterium smegmatis*. FEBS J. febs.15245. Available from: https://onlinelibrary.wiley.com/doi/abs/10.1111/febs.15245 10.1111/febs.15245 32037686

[msaa215-B27] Hecht N , Regev O, Dovrat D, Aharoni A, Gur E. 2018. Proteasome accessory factor A (PafA) transferase activity makes sense in the light of its homology with glutamine synthetase. J Mol Biol. [Internet] 430(5):668–681. 10.1016/j.jmb.2018.01.009 29397952

[msaa215-B28] Hug LA , Baker BJ, Anantharaman K, Brown CT, Probst AJ, Castelle CJ, Butterfield CN, Hernsdorf AW, Amano Y, Ise K. 2016. A new view of the tree of life. Nat Microbiol. [Internet] 1:16048. 10.1038/nmicrobiol.2016.48 27572647

[msaa215-B29] Iyer LM , Abhiman S, Maxwell Burroughs A, Aravind L. 2009. Amidoligases with ATP-grasp, glutamine synthetase-like and acetyltransferase-like domains: synthesis of novel metabolites and peptide modifications of proteins. Mol Biosyst. 5(12):1636. 20023723 10.1039/b917682a PMC3268129

[msaa215-B30] Iyer LM , Burroughs AM, Aravind L. 2008. Unraveling the biochemistry and provenance of pupylation: a prokaryotic analog of ubiquitination. Biol Direct 3(1):45. 18980670 10.1186/1745-6150-3-45 PMC2588565

[msaa215-B31] Kaltenbach M , Tokuriki N. 2014. Dynamics and constraints of enzyme evolution. J Exp Zool (Mol Dev Evol.) 322(7):468–487. 10.1002/jez.b.22562 24522979

[msaa215-B32] Kalyaanamoorthy S , Minh BQ, Wong TKF, von Haeseler A, Jermiin LS. 2017. ModelFinder: fast model selection for accurate phylogenetic estimates. Nat Methods 14(6):587–589. 28481363 10.1038/nmeth.4285 PMC5453245

[msaa215-B33] Katoh K , Standley DM. 2013. MAFFT multiple sequence alignment software version 7: improvements in performance and usability. Mol Biol Evol. [Internet] 30(4):772–780. 10.1093/molbev/mst010 PMC3603318 23329690

[msaa215-B34] Landau M , Mayrose I, Rosenberg Y, Glaser F, Martz E, Pupko T, Ben-Tal N. 2005. ConSurf 2005: the projection of evolutionary conservation scores of residues on protein structures. Nucleic Acids Res. [Internet] 33(Web Server):W299–302. 10.1093/nar/gki370 PMC1160131 15980475

[msaa215-B35] Lerner CG , Inouye M. 1990. Low copy number plasmids for regulated low-level expression of cloned genes in *Escherichia coli* with blue/white insert screening capability. Nucleic Acids Res. [Internet] 18(15):4631. 10.1093/nar/18.15.4631 PMC331321 2201955

[msaa215-B36] Maynard Smith J. 1970. Natural selection and the concept of a protein space. Nature [Internet] 225(5232):563–564. 10.1038/225563a0 5411867

[msaa215-B37] Meng EC , Pettersen EF, Couch GS, Huang CC, Ferrin TE. 2006. Tools for integrated sequence–structure analysis with UCSF Chimera. BMC Bioinformatics 7(1):1–10. 16836757 10.1186/1471-2105-7-339 PMC1570152

[msaa215-B38] Mistry J , Finn RD, Eddy SR, Bateman A, Punta M. 2013. Challenges in homology search: HMMER3 and convergent evolution of coiled-coil regions. Nucleic Acids Res. [Internet] 41(12):e121–e121. 10.1093/nar/gkt263 PMC3695513 23598997

[msaa215-B39] Nguyen L-T , Schmidt HA, von Haeseler A, Minh BQ. 2015. IQ-TREE: A fast and effective stochastic algorithm for estimating maximum-likelihood phylogenies. Mol Biol Evol. [Internet] 32(1):268–274. 10.1093/molbev/msu300 PMC4271533 25371430

[msaa215-B40] Özcelik D , Barandun J, Schmitz N, Sutter M, Guth E, Damberger FF, Allain FH-T, Ban N, Weber-Ban E. 2012. Structures of Pup ligase PafA and depupylase Dop from the prokaryotic ubiquitin-like modification pathway. Nat Commun. [Internet] 3:1014. 10.1038/ncomms2009 PMC4351746 22910360

[msaa215-B41] Pearce MJ , Mintseris J, Ferreyra J, Gygi SP, Darwin KH. 2008. Ubiquitin-like protein involved in the proteasome pathway of *Mycobacterium tuberculosis*. Science (80-.) [Internet] 322(5904):1104–1107. 10.1126/science.1163885 PMC2698935 18832610

[msaa215-B42] Pettersen EF , Goddard TD, Huang CC, Couch GS, Greenblatt DM, Meng EC, Ferrin TE. 2004. UCSF Chimera—a visualization system for exploratory research and analysis. J Comput Chem. 25(13):1605–1612. 15264254 10.1002/jcc.20084

[msaa215-B43] Regev O , Korman M, Hecht N, Roth Z, Forer N, Zarivach R, Gur E. 2016. An extended loop of the Pup ligase, PafA, mediates interaction with protein targets. J Mol Biol. [Internet] 428(20):4143–4153. 10.1016/j.jmb.2016.07.021 27497689

[msaa215-B44] Salisbury FB. 1969. Natural selection and the complexity of the gene. Nature [Internet] 224(5217):342–343. 10.1038/224342a0 5343878

[msaa215-B45] Shannon P , Markiel A, Ozier 2 O, Baliga NS, Wang JT, Ramage D, Amin N, Schwikowski B, Ideker T. 2003. Cytoscape: a software environment for integrated models of biomolecular interaction networks. Genome Res. 13(11):2498–2504. 14597658 10.1101/gr.1239303 PMC403769

[msaa215-B46] Sievers F , Wilm A, Dineen D, Gibson TJ, Karplus K, Li W, Lopez R, McWilliam H, Remmert M, Söding J, et al 2011. Fast, scalable generation of high-quality protein multiple sequence alignments using Clustal Omega. Mol Syst Biol. 7:539. 21988835 10.1038/msb.2011.75 PMC3261699

[msaa215-B47] Smoot ME , Ono K, Ruscheinski J, Wang P-L, Ideker T. 2011. Cytoscape 2.8: new features for data integration and network visualization. Bioinformatics [Internet] 27(3):431–432. 10.1093/bioinformatics/btq675 PMC3031041 21149340

[msaa215-B48] Stover CK , de la Cruz VF, Fuerst TR, Burlein JE, Benson LA, Bennett LT, Bansal GP, Young JF, Lee MH, Hatfull GF, et al 1991. New use of BCG for recombinant vaccines. Nature [Internet] 351(6326):456–460. 10.1038/351456a0 1904554

[msaa215-B49] Striebel F , Imkamp F, Sutter M, Steiner M, Mamedov A, Weber-Ban E. 2009. Bacterial ubiquitin-like modifier Pup is deamidated and conjugated to substrates by distinct but homologous enzymes. Nat Struct Mol Biol. 16(6):647–651. 19448618 10.1038/nsmb.1597

[msaa215-B50] Troshin PV , Procter JB, Barton GJ. 2011. Java bioinformatics analysis web services for multiple sequence alignment-JABAWS: MSA. Bioinformatics 27(14):2001–2002. 21593132 10.1093/bioinformatics/btr304 PMC3129525

[msaa215-B51] Vallenet D , Calteau A, Cruveiller S, Gachet M, Lajus A, Josso A, Mercier J, Renaux A, Rollin J, Rouy Z, et al 2017. MicroScope in 2017: an expanding and evolving integrated resource for community expertise of microbial genomes. Nucleic Acids Res. 45(D1):D517–528. 27899624 10.1093/nar/gkw1101 PMC5210572

[msaa215-B52] Vallenet D , Engelen S, Mornico D, Cruveiller S, Fleury L, Lajus A, Rouy Z, Roche D, Salvignol G, Scarpelli C, et al 2009. MicroScope: a platform for microbial genome annotation and comparative genomics. Database [Internet] 2009:bap021. 10.1093/database/bap021 PMC2790312 20157493

[msaa215-B53] Waterhouse AM , Procter JB, Martin DMA, Clamp M, Barton GJ. 2009. Jalview Version 2—a multiple sequence alignment editor and analysis workbench. Bioinformatics [Internet]. 25(9):1189–1191. 10.1093/bioinformatics/btp033 PMC2672624 19151095

