# Supplementary material for: Exploring Protein Space: From Hydrolase to Ligase by Substitution
Source: Mol Biol Evol. 2020 Sep 1;38(3):761–76. doi: 10.1093/molbev/msaa215 (PMC7947786; doi:10.1093/molbev/msaa215)
Supplement: msaa215_Supplementary_Data [file msaa215_supplementary_data.zip › Supplementary Figs.pdf]

Fig. S1

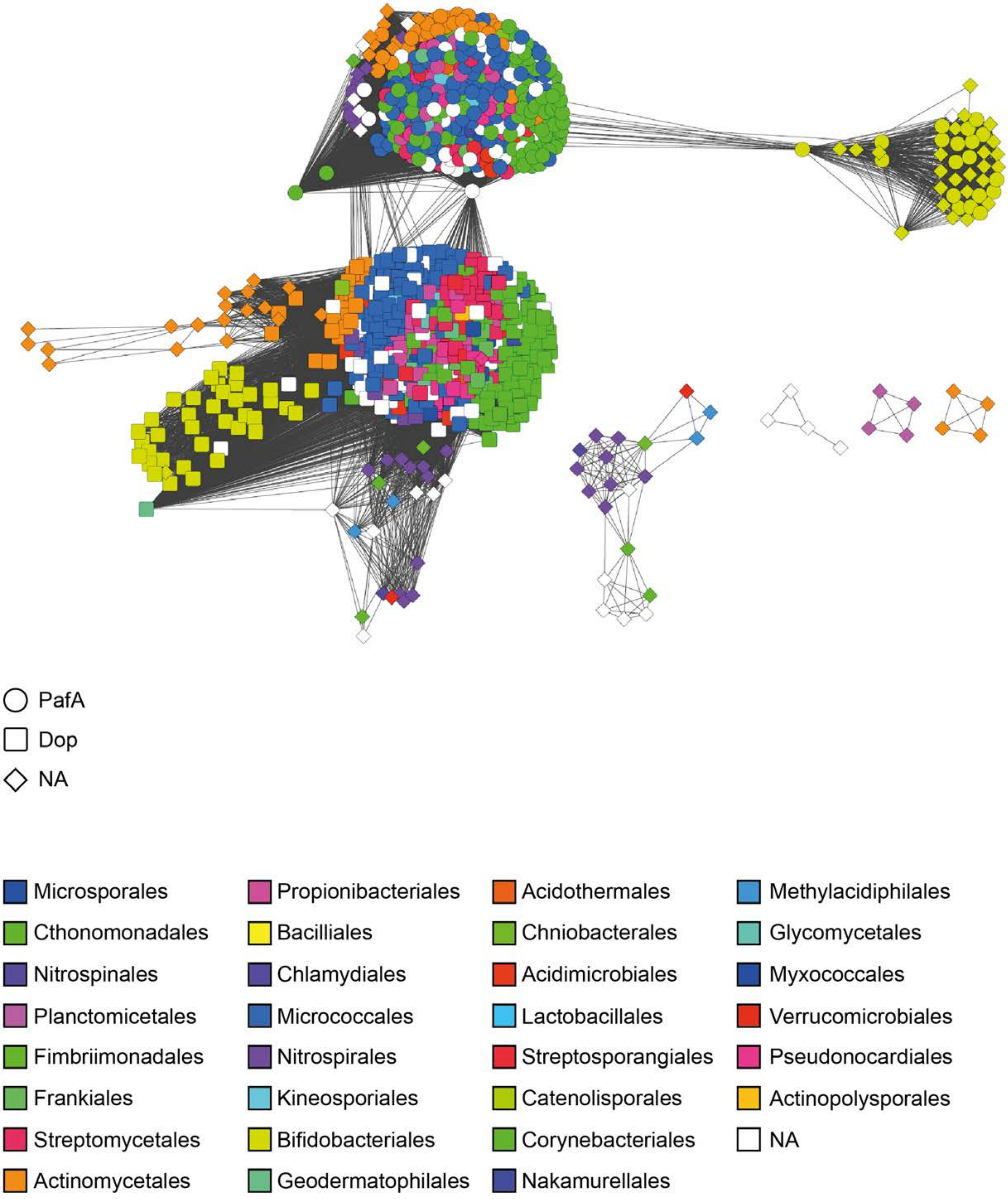
PafA
Dop
NA
Microsporales
Cthonomonadales
Nitrospinales
Planctomicetales
Fimbriimonadales
Frankiales
Streptomycetales
Actinomycetales
Propionibacteriales
Bacilliales
Chlamydiales
Micrococcales
Nitrospirales
Kineosporiales
Bifidobacteriales
Geodermatophilales
Acidothermales
Chniobacterales
Acidimicrobiales
Lactobacillales
Streptosporangiales
Catenolisporales
Corynebacteriales
Nakamurellales
Methylacidiphilales
Glycomycetales
Myxococcales
Verrucomicrobiales
Pseudonocardiales
Actinopolysporales
NA

Fig. S2

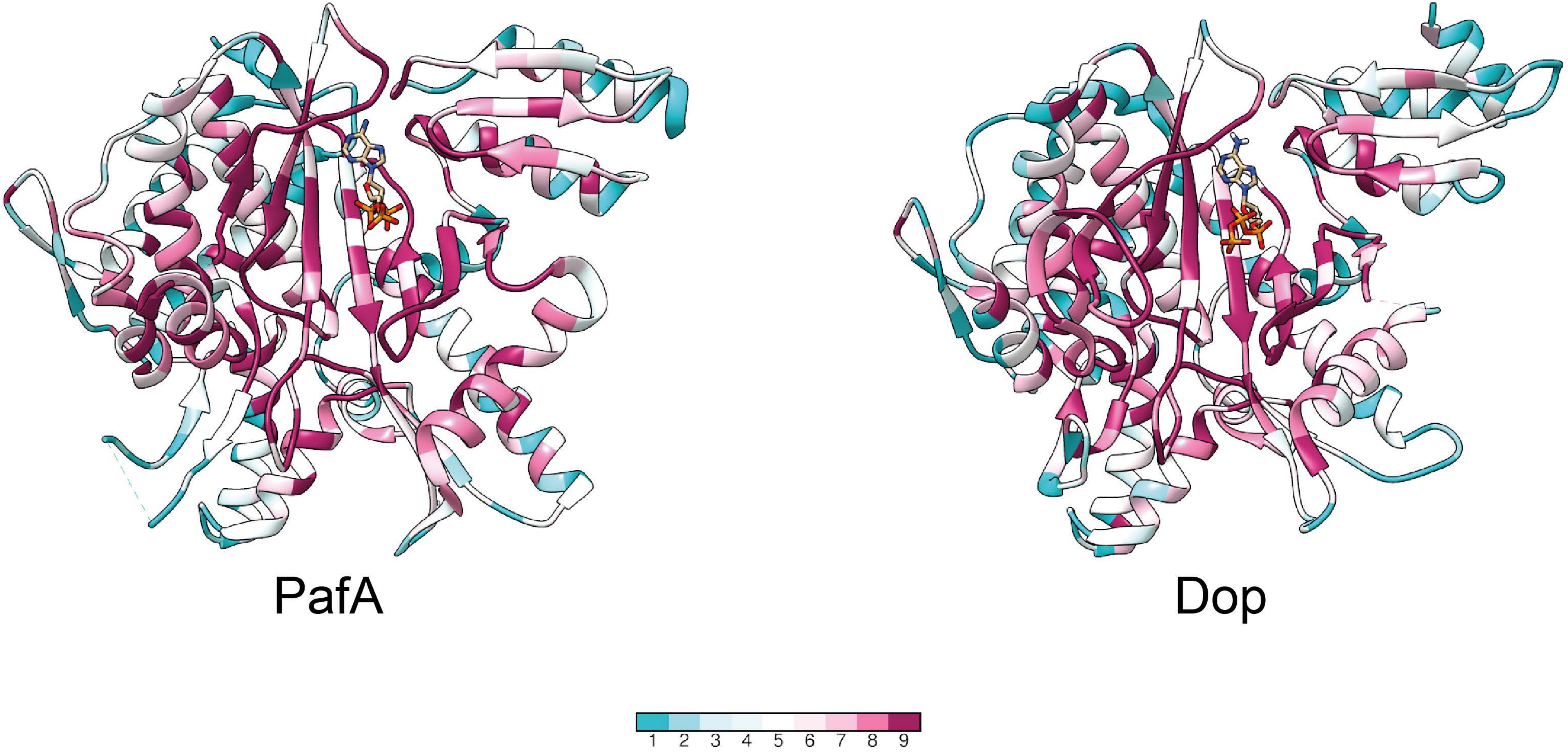

Fig. S3

A.

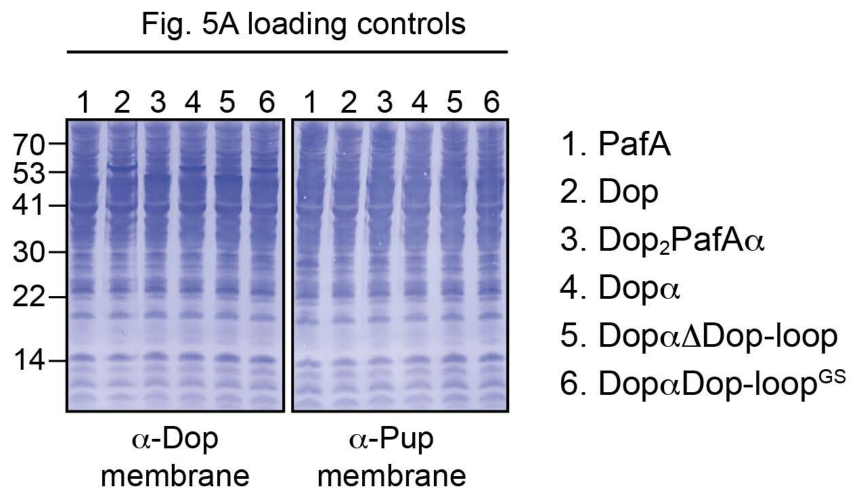
Fig. 5A loading controls
1 2 3 4 5 6 1 2 3 4 5 6
70
53
41
30
22
14
α-Dop membrane
α-Pup membrane
1. PafA
2. Dop
3. Dop2PafAα
4. Dopα
5. DopαΔDop-loop
6. DopαDop-loopGS

B.

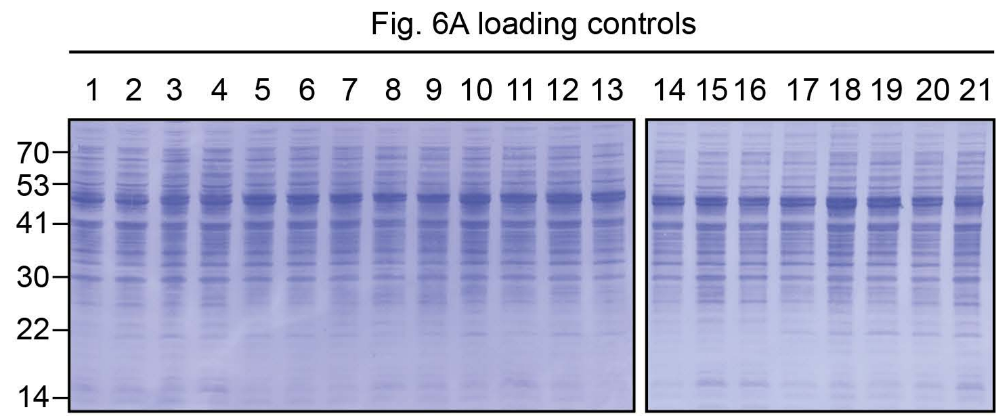
Fig. 6A loading controls
1 2 3 4 5 6 7 8 9 10 11 12 13 14 15 16 17 18 19 20 21
70
53
41
30
22
14

B.

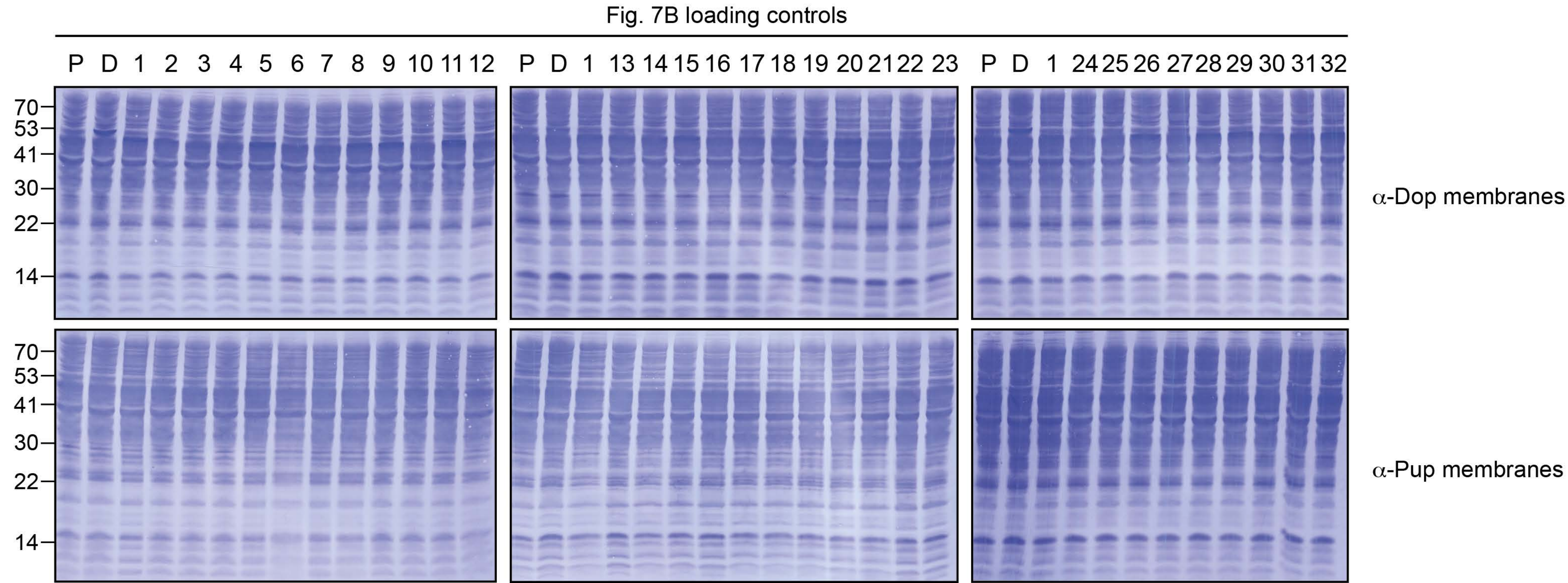
Fig. 7B loading controls
P D 1 2 3 4 5 6 7 8 9 10 11 12 P D 1 13 14 15 16 17 18 19 20 21 22 23 P D 1 24 25 26 27 28 29 30 31 32
70
53
41
30
22
14
α-Dop membranes
α-Pup membranes
